# Development of a vegetation canopy reflectance sensor and its diurnal applicability under clear sky conditions

**DOI:** 10.3389/fpls.2024.1512660

**Published:** 2025-01-09

**Authors:** Naisen Liu, Jingyu Guo, Fuxia Liu, Xuedong Zha, Jing Cao, Yuezhen Chen, Haixia Yan, Chenggong Du, Xuqi Wang, Jiping Li, Yongzhen Zhao

**Affiliations:** ^1^ Jiangsu Collaborative Innovation Center of Regional Modern Agriculture & Environmental Protection, Huaiyin Normal University, Huai’an, China; ^2^ Jiangsu Key Laboratory for Eco-Agricultural Biotechnology around Hongze Lake, Huaiyin Normal University, Huai’an, China; ^3^ Jiangsu Engineering Research Center for Cyanophytes Forecast and Ecological Restoration of Hongze Lake, Huaiyin Normal University, Huai’an, China; ^4^ Huai’an Agricultural Information Center, Huai’an, China; ^5^ Jiangsu Academy of Agricultural Sciences, Wuxi, China; ^6^ Huai’an Institute of Vegetable Sciences, Huai’an, China; ^7^ Huai’an Agricultural Technology Extension Center, Huai’an, China

**Keywords:** vegetation canopy reflectance, spectral reflectance, adaptability to varying light intensities, solar altitude correction, stability of full-daytime measurements

## Abstract

The spectral reflectance provides valuable information regarding vegetation growth and plays an important role in agriculture, forestry, and grassland management. In this study, a small, portable vegetation canopy reflectance (VCR) sensor that can operate throughout the day was developed. The sensor includes two optical bands at 710 nm and 870 nm, with the light separated by filters, and has a field of view of 28°. It is powered by two 14500 rechargeable batteries and uses Wi-Fi for data transmission. The calibration of the sensor was performed using an integrating sphere, and a solar altitude correction model was constructed. The sensor’s accuracy was validated using a standard reflectance gray scale board. The results indicate that the root mean square error (RMSE) and mean absolute error (MAE) at 710 nm were 1.07% and 0.63%, respectively, while those at 870 nm were 0.94% and 0.50%, respectively. Vegetation at 14 sites was measured using both the VCR sensor and an Analytical Spectral Devices (ASD) spectroradiometer at nearly the same time for each site. The results show that the reflectance values measured by both devices were closely aligned. Measurements of Bermuda grass vegetation on clear days revealed that the intra-day reflectance range at 710 nm narrowed from 12.3–19.2% before solar altitude correction to 11.1–13.4% after correction, and the coefficient of variation (CV) decreased from 10.86% to 2.93%. Similarly, at 870 nm, the intra-day reflectance range decreased from 41.6–60.3% to 39.0–42.0%, and the CV decreased from 9.69% to 1.53%. In summary, this study offers a fundamental tool for monitoring vegetation canopy reflectance in the field, which is crucial for advancing high-quality agricultural, grassland, and forest management practices.

## 1 Introduction

The vegetation canopy reflectance can be used to obtain vegetation growth information such as the chlorophyll content, biomass, and leaf area index. Hence, it plays an important role in agriculture, forestry, and grassland management (Wang et al., 2012; Zulfa et al., 2020; Zhao et al., 2021; Galal et al., 2022). The reflectance spectrum of the vegetation canopy is influenced by factors such as the leaf area index, biomass, chlorophyll content, and nitrogen content. Therefore, by measuring the canopy’s spectral reflectance in specific bands, valuable information about vegetation growth can be obtained. Instruments capable of collecting data over a wide spectral band have promise in advanced research on vegetation spectral monitoring, such as the FieldSpec spectroradiometers (Analytical Spectral Devices Inc., Boulder, CO, USA). The FieldSpec FR has a spectral range of 350–2500 nm, while the FieldSpec HandHeld has a spectral range of 325–1075 nm, and both devices have a spectral resolution of 1 nm. Due to its ability to capture detailed vegetation spectral characteristics, it has been widely used to monitor vegetation growth, including parameters such as the nitrogen content (Liu et al., 2020; Smith et al., 2020), biomass (Pradhan et al., 2014; Zhang et al., 2018), chlorophyll content (Li et al., 2020; Morley et al., 2020), and sugar content (Zhang et al., 2015). It has also been used to monitor the aging process (Anderegg et al., 2020), disease (Zhao et al., 2012; Martínez-Martínez et al., 2018), and water stress (Sönmez et al., 2008). The FieldSpec spectroradiometer uses sunlight as its light source and requires calibration with a white target board before measurement. To ensure accurate reflectance measurements, frequent calibration is necessary, especially when the light intensity changes rapidly, which can greatly reduce the working efficiency. Multispectral radiometers (MSR, Cropscan, Rochester, MN, USA) also use sunlight as the light source, but they can simultaneously measure the incident sunlight and canopy-reflected light to obtain the reflectance (Zhu et al., 2007; Gianquinto et al., 2011). The spectral range includes the visible and near-infrared bands. The MSR16R has 16 bands, which can be selected from 116 bands within the range of 460–1700 nm. The MSR87 has eight bands with central wavelengths of 460, 510, 560, 610, 660, 710, 760, and 810 nm. The MSR5 has five bands with central wavelengths of 485, 560, 660, 830, and 1650 nm. Although the FieldSpec spectroradiometer and MSR offer rich spectral bands, their high costs and large sizes make them unsuitable for use in agricultural production.

Based on previous research on spectral technologies (e.g., sensitive bands and inversion models) for monitoring vegetation growth, developing low-cost specialized sensors with only the necessary sensitive bands is of great significance for widespread application. Wang et al. (2004) developed a portable normalized difference vegetation index (NDVI) instrument for estimating the growth of winter wheat, utilizing spectral bands in the red and near-infrared regions. Measurements of winter wheat demonstrated that the NDVI values exhibited minimal variation around midday, making midday the optimal time for data collection. Cui et al. (2009) developed an optical sensor for measuring the crop leaf chlorophyll content. This sensor obtains the reflectance by measuring the light intensities of the canopy-reflected light and incident sunlight at 610 and 1220 nm, and it then calculates the NDVI based on the reflectance. Subsequently, the chlorophyll content is derived from the NDVI. The NDVI values of the white reference panel were measured from 10:00 to 15:40, and the results indicated that the NDVI values were relatively stable between 10:00 and 14:40, but they exhibited a sharp decline after 14:40. Ni et al. (2018) designed a portable apparatus for crop-growth monitoring and diagnosis (CGMD). It measures the spectral reflectance of rice and wheat canopies at 720 and 810 nm, and the leaf nitrogen content (LNC), leaf nitrogen accumulation (LNA), leaf area index (LAI), and leaf dry weight (LDW) can be calculated. The intensity of skylight varies significantly across different seasons and throughout the day, potentially impacting the accuracy of sensor measurements. However, for most existing sensors, there is a lack of studies on their adaptability to both strong and weak light conditions. Newly developed sensors require thorough evaluation of their all-day measurement accuracies to ensure their suitability for practical applications in production.

Differences in ambient light can affect the accuracy of spectral sensors that use sunlight as a light source (Shibayama and Wiegand, 1985). The position of the sun, primarily the solar altitude or zenith angle, affects ambient light, especially the angle of the sunlight. Throughout the day, the solar altitude first increases, reaches a peak at noon, and then slowly decreases. The solar zenith angle, which is complementary to the solar altitude, exhibits the opposite trend. When the sensor’s optical window is facing downward (i.e., view zenith = 0°) to receive reflected light from the ground, the measured reflectance is called the nadir reflectance, and the reflectance measured at other view zeniths is called the off-nadir reflectance. In most practical applications, the sensor measures the nadir reflectance. Rijks (1967) measured the all-day nadir reflectance of cotton on multiple dates and found that the nadir reflectance decreased as the solar altitude increased, that is, a U-shaped diurnal variation pattern with the smallest values observed at noon. Davies and Buttimor (1969) reported similar diurnal variations in the reflectance of peppers, tobacco, perennial ryegrass, tomatoes, winter wheat, and corn. Proctor et al. (1972) showed that the diurnal variations in the reflectance of apple trees depended on the solar zenith angle, but the seasonal variations were small. Similar findings have been reported in other studies (Ranson et al., 1985; Shikada and Miyakita, 1992), indicating that the solar altitude impacts the vegetation reflectance. Kimes et al. (1980) determined the relationship between the reflectance and the vegetation canopy characteristics and solar zenith angle. Ranson et al. (1985) attributed such reflectance variations to specular reflection caused by direct sunlight. The influence of solar altitude on the measurement accuracy of sensors poses a significant challenge for all-day vegetation spectral reflectance monitoring.

To reduce the influence of the solar altitude on the spectral reflectance, the optical window of the sensor is often equipped with a cosine corrector. The light passing through the cosine corrector is diffuse and non-directional. The cosine corrector is often constructed from opal glass or polytetrafluoroethylene (Ni et al., 2018; Liu et al., 2022; Bai et al., 2023; Heusinkveld et al., 2023). However, assessments of the extent to which a cosine corrector can reduce the impact of the solar altitude angle on reflectance measurements, especially throughout the day, are extremely scarce. To minimize the impact of the solar altitude on vegetation canopy reflectance measurements, data collection is typically performed around midday (e.g., 10:00–14:00 or 11:00–13:00) when changes in the solar altitude are minimal (Tian et al., 2005; Huang et al., 2007; Zhang et al., 2018; Anderegg et al., 2020; Chandel et al., 2021). However, this approach reduces the data collection time each day, and the high temperatures around noon in summer are not suitable for fieldwork.

To address the aforementioned typical issues, in this study, we developed a novel portable sensor for measuring the vegetation canopy reflectance (VCR). The sensor features a photodetection circuit with 250 gain levels, providing a wide dynamic range that effectively adapts to variations in the ambient light intensity. Additionally, the sensor is equipped with a resistance calibration circuit, ensuring a high reflectance measurement accuracy. A solar elevation correction model was constructed based on the characteristics of optical transmission, enabling the sensor to operate reliably throughout the day under clear weather conditions.

## 2 Materials and methods

In this section, we detail the design process of the VCR sensor, as well as the experiments conducted to evaluate its accuracy and stability in measuring vegetation spectral reflectance.

### 2.1 Design of the VCR sensor

**Figure 1:**
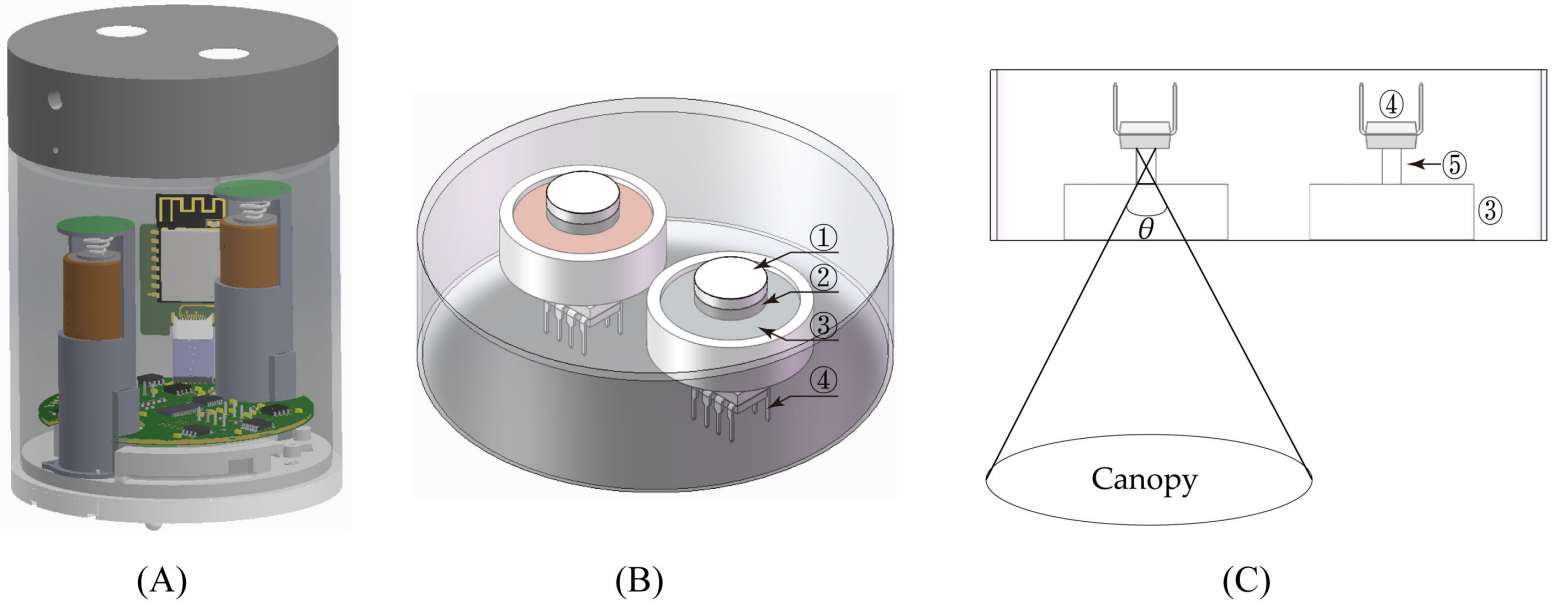
Schematic diagrams of **(A)** the VCR sensor, **(B)** the up-facing optical sensing unit, and **(C)** the down-facing optical sensing unit with the field of view. ① Cosine corrector, ② Attenuator, ③ Filter, ④ Photodetector, and ⑤ Optical channel.

The VCR sensor consists of mechanical components, optical systems, hardware circuits, power supplies, and software systems; the overall structure of the sensor is illustrated in **Figure 1A**. The sensor uses Wi-Fi for data transmission.

#### 2.1.1 Optical sensing unit

The spectral reflectance of the vegetation canopy is defined as the ratio of the light energy reflected by the canopy in a specific band to the incident light energy in that band. The up-facing optical sensing unit of the VCR sensor captures incident light, while the down-facing optical sensing unit detects light reflected from the vegetation canopy. Given that the vegetation canopy receives light from the entire sky, the up-facing optical sensing unit has a 180°FOV. On sunny days, the light from the sky predominantly consists of direct sunlight (Liu and Jordan, 1960; Sen, 2004). The solar altitude influences the transmittance of the direct sunlight entering the sensor, thereby affecting the accuracy of the reflectance measurements. The optical window of the up-facing optical sensing unit is equipped with a cosine corrector made of polytetrafluoroethylene (PTFE), which could reduce the impact of the solar altitude on the measurements. Moreover, the cosine corrector ensures that the up-facing optical sensing unit maintains a 180°FOV. Additionally, an attenuator, a filter, and a photodetector are installed below the cosine corrector (**Figure 1B**). The down-facing optical sensing unit is equipped with filters and photodetectors from bottom to top. The FOV significantly influences the area of vegetation measured by the sensor when capturing spectral information, thereby affecting the measurement accuracy. A larger FOV covers a wider vegetation area, enabling the spectral information to better represent the overall vegetation conditions. However, in areas with incomplete vegetation coverage, particularly for row-planted crops, an excessively large FOV may capture spectral information about both the vegetation and soil, leading to inaccurate measurements. Conversely, a smaller FOV can effectively reduce or eliminate soil interference, but it may result in measurements that are not representative of the vegetation. Therefore, the FOV must be carefully balanced. Currently, an FOV of approximately 30° is commonly used. The VCR sensor is designed with an FOV of 28°. A schematic diagram of the FOV is shown in **Figure 1C**.

The filters used are FB710-10 and FB870-10 (Thorlabs Inc., Newton, NJ, USA), which have wavelengths of 710 nm and 870 nm, respectively, and a full width at half-maximum (FWHM) of 10 nm. The photodetector converts the optical signal into an electrical signal, and its spectral responsivity determines the performance of the sensor. The VCR sensor uses OPT101 photodiodes (Texas Instruments, Dallas, TX, USA), which have a spectral responsivity of 0.5–0.6 A/W at 710 nm and 870 nm. The photosensitive surface of the OPT101 measures 2.29 mm × 2.29 mm. The OPT101 photodiode operates in photoconductive mode, resutling in excellent linearity and a low dark current.

#### 2.1.2 Design of the hardware circuit

The sensor’s circuitry primarily consists of a power module, optical signal acquisition module, calibration module for the amplifier circuit resistance, and wireless communication module. The power module, wireless communication module, and up-facing optical signal acquisition module each has its own printed circuit board (PCB), while the down-facing optical signal acquisition module and microcontroller unit (MCU) are on the same PCB. This split design facilitates sensor installation and maintenance.

**Figure 2:**
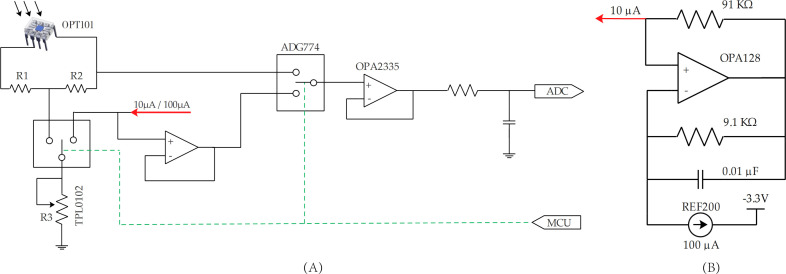
**(A)** Schematic diagram of the optical signal acquisition and TPL0102 resistor calibration circuit, and **(B)** schematic diagram of the 10 µA circuit.


The power module utilizes two 3.7 V 14500 batteries connected in parallel to supply 3.3 V and −3.3 V power to the system. The OPT101 photodetector has an integrated operational amplifier that enables current-to-voltage (I–V) conversion, with outputs from pin 5. Different gains can be achieved by connecting an external feedback resistor between pins 2 and 5. The VCR sensor is intended for year-round operation. Given that the ambient light intensity varies significantly across the seasons, it is necessary to dynamically adjust the gain so as to obtain an appropriate voltage. The VCR sensor employs a T-type feedback network to achieve a high amplification factor using three small resistors (**Figure 2A**). R1 and R2 are precision resistors with a resistance of 68 kΩ and an accuracy of 0.01%. R3 is replaced by a digital potentiometer TPL0102 (Texas Instruments, Dallas, TX, USA), which has 256 taps and a resistance range of 0–100 kΩ. A larger resistor number indicates a higher resistance value. A smaller resistance value of R3 means a greater gain of the OPT101 (Equation 1). The accuracy of R3 directly affects the gain of the OPT101 and consequently the accuracy of the sensor’s reflectance measurement. Therefore, it is essential to calibrate the different resistance values of the TPL0102. Specifically, the TPL0102 is connected to the feedback network and a constant current source through an analog multiplexer, namely, ADG774 (Analog Devices, MA, USA), to achieve resistance calibration (**Figure 2A**). Due to the limited FOV and weak light reflected from the canopy, the optical signal received by the down-facing optical sensing unit is very weak and requires a higher gain. Based on preliminary testing under both strong and low-light conditions, the required gain ranges approximately from 1 M to 6 M. Hence, only resistors 1–25 of the TPL0102 are calibrated. The up-facing optical sensing unit receives strong light from the sky, so it requires only a small amount of amplification. The minimum required gain is approximately 0.2 M, as determined through initial testing. Hence, resistors 1–250 are calibrated. In the down-facing optical signal acquisition circuit, the TPL0102 is connected to a 100 µA constant current source, while the up-facing TPL0102 is connected to a 10 µA constant current source. The 100 µA current is provided by REF200 (Texas Instruments, Dallas, TX, USA), and the 10 µA current is provided by REF200 and OPA128 (Texas Instruments, Dallas, TX, USA) (**Figure 2B**). The on-resistance of ADG774 is 2.2 Ω, which is minimal and has a negligible impact on the other circuits during operation. The MCU uses the 16-bit MSP430I2041 (Texas Instruments, Dallas, TX, USA) for signal acquisition control, data calculation, and communication. The MSP430I2041 features a low supply voltage (2.2–3.6 V), ultra-low power consumption, five power-saving modes, and four 24-bit analog-to-digital converters (ADCs), making it suitable for portable devices and equipment used in field operations. The VCR sensor utilizes the MSP430I2041’s four built-in ADCs to convert analog electrical signals, which represent the light intensity, into digital signals. The sensor uses the ESP-12S (Ai-thinker Technology Co., Shenzhen, China) for wireless communication.

(1) $$G=68+68+\frac{68\times 68}{R}$$

where G is the amplification (K); and *R* is the resistance value after calibration of the TPL0102 (kΩ). The calibration method is described in Section 2.2.1.

#### 2.1.3 Design of the VCR sensor control system

**Figure 3:**
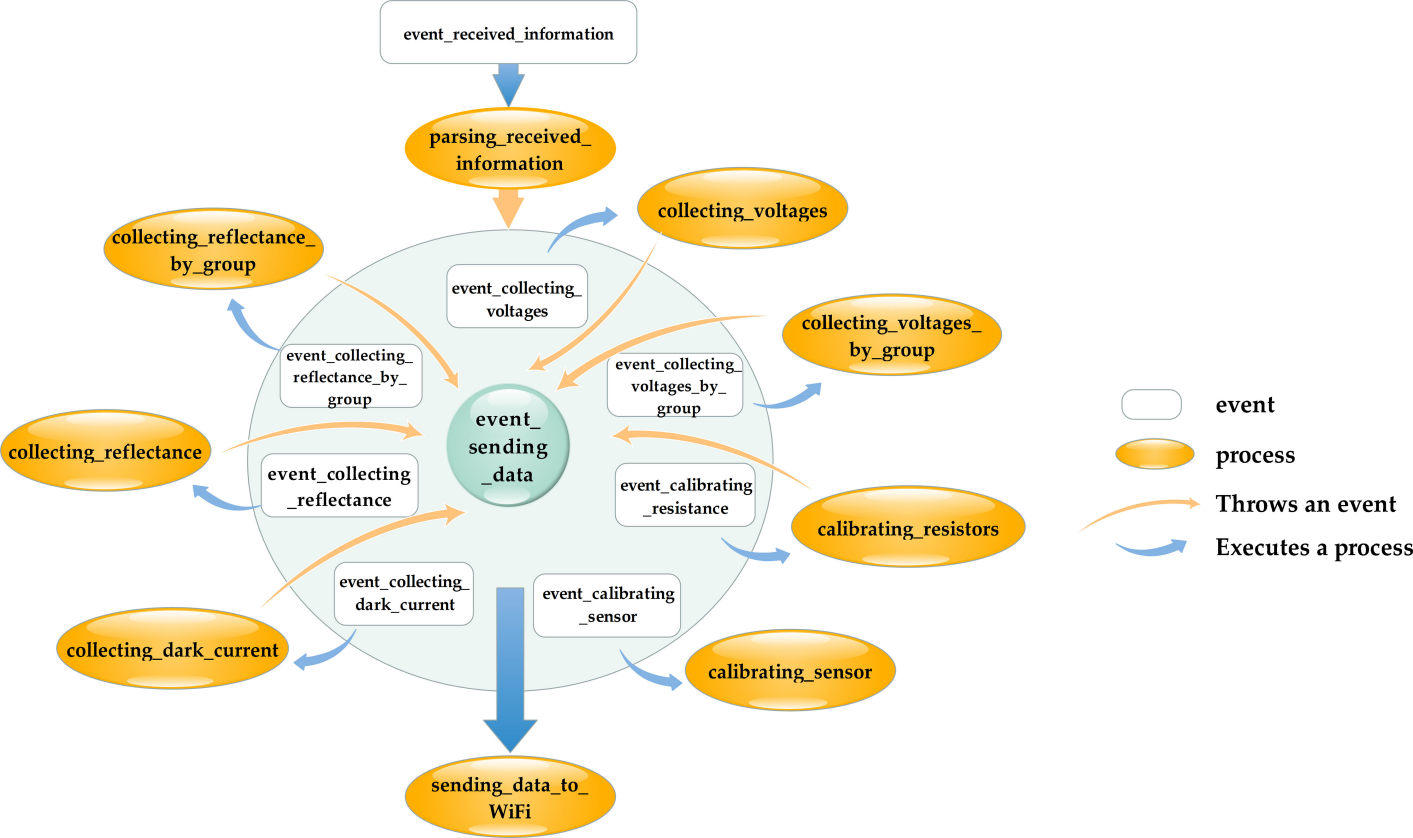
Schematic diagram of the system events and processes.

The embedded software system of the VCR sensor handles the measurement and communication tasks. The software was developed using the Contiki embedded operating system, which is event-driven and supports multithreading. The Contiki requires minimal resources during the run period, making it highly suitable for sensor applications (Dunkels et al., 2004). Upon powering on, the VCR sensor initializes, and then, it enters a sleep state and waits for events to occur. The events and processes of this system are shown in **Figure 3**. The events include *event_received_information, event_collecting_reflectance, event_collecting_reflectance_by_group*, *event_collecting_voltages, event_collecting_voltages_by_group, event_calibrating_resistance, event_calibrating_sensor, event_collecting_dark_current*, and *event_sending_data*. The system processes include *parsing_received_information, collecting_reflectance, collecting_reflectance_by_group, collecting_voltages, collecting_voltages_by_group, calibrating_resistors, calibrating_sensor, collecting_dark_current*, and *sending_data_to_WiFi*.

The user group for this sensor includes staff who measure the spectral reflectance of the vegetation canopies and scientific researchers. To facilitate their work, a command set was designed, which includes the following commands: “COLLECT_Ref” for collecting the reflectance, “COLLECT_Ref Repeat[]” for collecting the reflectance by group, “COLLECT_Voltages” for collecting the voltages of the optical channels, “COLLECT_Voltages Repeat[]” for collecting the voltages of optical channels by group, “Calibrate_Resistors” for calibrating the resistors of TPL0102, “Calibrate_Sensor” for calibrating the reflectance of the sensor, and “Dark_Current” for collecting each channel’s dark current. The sensor’s temperature is also recorded during each measurement. The commands “COLLECT_Ref Repeat[]” and “COLLECT_Voltages Repeat[]” enable multiple measurements to be obtained using a single command. The brackets can be filled with multiple numbers separated by commas, each of which represents the number of sampling times during the measurement. For example, [1,5,10] means three measurements are required: the first is a single measurement, the second measurement is taken five times consecutively and the average is taken as the result, and the third measurement is taken ten times consecutively with the average as the result. Similarly, [5,5,5,5] indicates four measurements, each taken five times consecutively, and the averages are taken as the results. This type of command enables repeated measurements within a short time frame, which is particularly useful for studying the impact of the wind speed on the vegetation canopy reflectance.

Devices such as mobile phones and computers can connect to the sensor via Wi-Fi and control it by sending commands, and the sensor will send back the measured data. Upon receiving a command, the *parsing_received_information* process parses the content and triggers the corresponding event based on the command. Once the event is triggered, the corresponding process will run. For measurement commands, the *event_sending_data* event is triggered after the data are measured, and the *sending_data_to_WiFi* process sends the data back.

**Figure 4:**
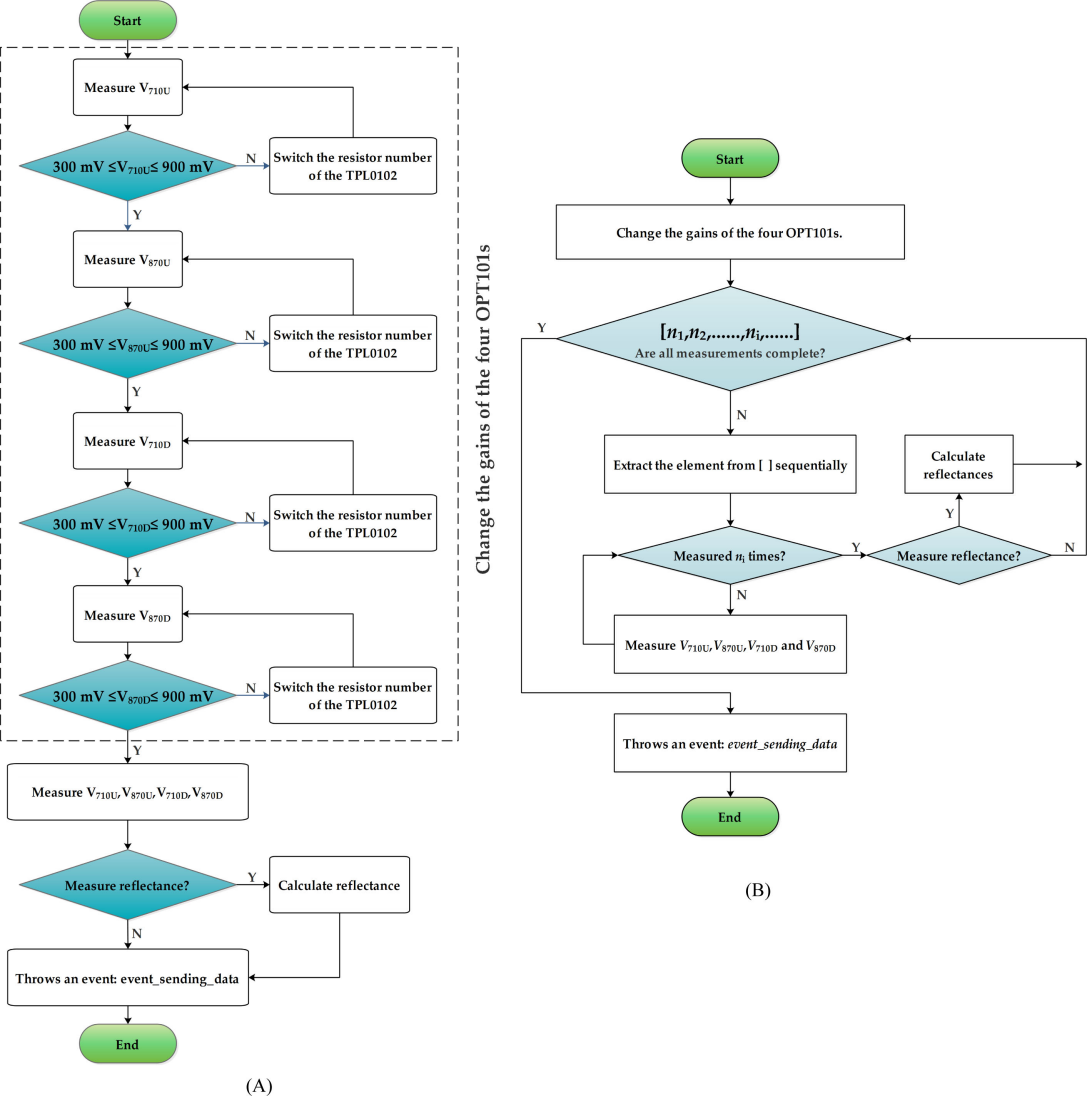
Procedure **(A)** for reflectance value and voltage measurement, and **(B)** that for reflectance value and voltage measurement by group.

**Figure 4** illustrates the processes for measuring the reflectance or voltages, as well as for measuring the reflectance or voltages by group. When measuring the reflectance or voltages (**Figure 4A**), the sensor first measures the voltage (V_710U_) of the 710 nm up-facing optical sensing unit. If the voltage is less than 300 mV, the resistance of the TPL0102 is reduced to increase the gain of the OPT101. If the voltage exceeds 900 mV, the gain of the OPT101 is reduced. After multiple measurements, the final voltage (*V*_710U_) is $300mV\leq V_{710U}\leq 900mV$ . Subsequently, the gains of the 870 nm up-facing optical sensing unit and the 710 nm and 870 nm down-facing optical units are adjusted. The voltages of all four optical units are then re-measured. If reflectance measurements are required, the reflectance is calculated. Finally the *event_sending_data* event is triggered.

When measuring by group (**Figure 4B**), the gains of all of the OPT101 optical units are first adjusted using the method illustrated in **Figure 4A**. After adjustment, the first number (*n*_1_) from the sequence [*n*_1_, *n*_2_, …, *n*_i_, …] is selected and *V*_710U_, *V*_870U_, *V*_710D_, and *V*_870D_ are measured successively *n*_1_ times. The average voltages or reflectance values are calculated and recorded as the result. Next, the second number (*n*_2_) is taken from the sequence, and *V*_710U_, *V*_870U_, *V*_710D_, and *V*_870D_ are measured *n*_2_ times. The average voltages or reflectance values are calculated and recorded again. This process is repeated until all of the numbers in the sequence are utilized. Finally, the *event_sending_data* event is triggered.

### 2.2 Research on calibration methods for the VCR sensor

The gain and dark current of each optical unit of the sensor affect the accuracy of the measurement, so the resistance of the TPL0102 potentiometer, the dark current of the OPT101, and the reflectivity of the sensor are calibrated.

#### 2.2.1 Calibration of the resistance values of the TPL0102

As can be seen from Equation 1, the accuracy of the TPL0102 resistance directly influences the gain of the OPT101. During calibration, the “Calibrate_Resistors” command was sent to the VCR sensor. The MCU then controlled the ADG774 (**Figure 2A**) to connect the TPL0102 chips within the 710 nm and 870 nm down-facing optical unit to a 100 µA current for calibration of resistors 1–25. The calibrated resistance value is denoted as *R*_D_. Similarly, the ADG774 was controlled to connected the TPL0102 chips within the 710 nm and 870 nm up-facing optical unit to a 10 µA current for calibration of resistors 1–250. The calibrated resistance value is denoted as *R*_U._ The calculation method for the resistance values is shown in Equations 2, 3.


(2) $$R_{U}=\frac{V}{10\mu A}=10V$$

(3) $$R_{D}=\frac{V}{10\mu A}=V$$

where *V* is the output voltage of the sensor (0.1 mV).

#### 2.2.2 Calibration of dark currents of the OPT101 in each optical unit

The VCR sensor was placed in a black, light-proof box. The command “Dark_Current” was sent to the sensor. The MCU controlled the ADG774 to connect the TPL0102 to the OPT101 circuit, and the resistor number of TPL0102 switched to adjust the gain of the OPT101. The dark current, expressed as the measured voltage, of each optical unit of the sensor at different gains was then measured.

#### 2.2.3 Calibration of the sensor reflectance

The sensor’s down-facing optical unit detects the light reflected from vegetation and converts it into voltage signal *U*_D_, while the up-facing optical unit detects incident light from the sky and converts it into voltage signal *U*_U_. The reflectance is calculated as the ratio $\rho =\frac{U_{D}}{U_{U}}$ . Calibration can be performed on the raw reflectance values, as $\rho =f\left(\frac{U_{D}}{U_{U}}\right)$ , where *f*() is calibration function. Alternatively, the voltages *U*_D_ and *U*_U_ can be calibrated separately, yielding a reflectance relationship of $\rho =\frac{f_{1}\left(U_{D}\right)}{f_{2}\left(U_{U}\right)}$ , where *f*_1_() and *f*_2_() are calibration functions. It is also possible to calibrate only one of the voltage signals, resulting in reflectance relationships such as $\rho =\frac{f_{1}\left(U_{D}\right)}{U_{U}}$ or $\rho =\frac{U_{D}}{f_{2}\left(U_{U}\right)}$ .

An integrating sphere was used to calibrate the VCR sensor’s output voltages, thereby calibrating the reflectance values. By adjusting the voltage of the integrating sphere’s light source, different light intensities were achieved. Under consistent light intensity conditions, the up-facing and down-facing optical windows of the sensor were aligned with the light outlet of the integrating sphere. The output voltage of the sensor was then measured, and the dark current was subtracted. Since the light intensity is uniform, the voltage of the down-facing optical unit in the same band should match that of the up-facing optical unit. Based on this principle, a voltage calibration equation f() was constructed to calibrate the reflectance. The reflectance was calibrated using Equation 4.


(4) $$\rho_{\lambda }=\frac{f\left(U_{D}/G_{D}\right)}{U_{U}/G_{U}}\times 100\%$$

where $\rho_{\lambda }$ is the reflectance at wavelength *λ*, *U_D_* and *G_D_* are respectively the measured voltage and gain of the down-facing optical unit at wavelength *λ*, *U_U_* and *G_U_* are the measured voltage and gain, respectively, of the up-facing optical unit at the same wavelength.

The accuracy of the reflectance measurement of the sensor was verified using a gray scale board with a reflectance of 30%. The experiment was conducted on the roof of the School of Life Sciences building at Huaiyin Normal University on August 4, 2024. The sensor was positioned approximately 17 cm above the 20 cm × 20 cm gray scale board. If the distance was greater than 17 cm, the sensor’s FOV would have extended beyond the gray scale board.

### 2.3 Experimental design for performance testing of the VCR sensor

#### 2.3.1 Accuracy testing experiment using standard reflectance gray boards

To verify the accuracy of the VCR sensor’s reflectance measurements, a total of seven gray scale boards were used as the measurement targets. The nominal reflectance values of the gray calibration panels were 10%, 20%, 25%, 40%, 60%, 80%, and 99%. However, the reflectance of the same gray panel varies across different spectral bands. According to the datasheets provided by the manufacturer, the reflectance values of the seven gray scale boards at the 710 nm wavelength are 11.5%, 18.8%, 24.3%, 40.2%, 61.5%, 79.2%, and 98.9%. At the 870 nm wavelength, the reflectance values are 11.8%, 18.0%, 23.0%, 38.3%, 61.2%, 79.1%, and 98.7%. The experiment was conducted from 14:57 to 15:06 on August 5, 2024, under clear sky conditions, during which the solar altitude decreased from 47.9° to 46.3°. The instrument was located on the roof of the School of Life Sciences building at Huaiyin Normal University. Each measurement was repeated ten times.

#### 2.3.2 Accuracy testing experiment using vegetation as the measurement subject

On a clear day, the VCR sensor and the ASD Fieldspec 4 spectroradiometer (ASD Inc., Alexandria, VA, USA) were used to simultaneously measure different vegetation canopies to further verify the accuracy of the VCR sensor’s vegetation reflectance measurements. A total of 14 vegetation sites were selected. Sites 1, 2, 4, 8, 10, 11, 12, and 14 contained *Imperata cylindrica* in varying growth stages or densities. Sites 3 and 9 consisted of *Goosegrass*, site 5 contained *Setaria viridis*, site 6 contained *Tall Fescue*, and sites 7 and 13 contained *Clover*. The FOV of the VCR sensor and the ASD spectroradiometer were 28° and 25°, respectively. To ensure consistent canopy coverage in both measurements, the VCR sensor and the ASD spectroradiometer’s fiber optic probe were located 40 cm and 48 cm above the measured vegetation, respectively. Given that the FWHM of the VCR sensor filter is 10 nm, the reflectance of the 705–715 nm band measured by the ASD spectroradiometer was averaged to represent the reflectance at 710 nm, and the average of the reflectance in the 865–875 nm band was taken as the reflectance at 870 nm.

#### 2.3.3 Accuracy testing for all-day measurement

**Figure 5:**
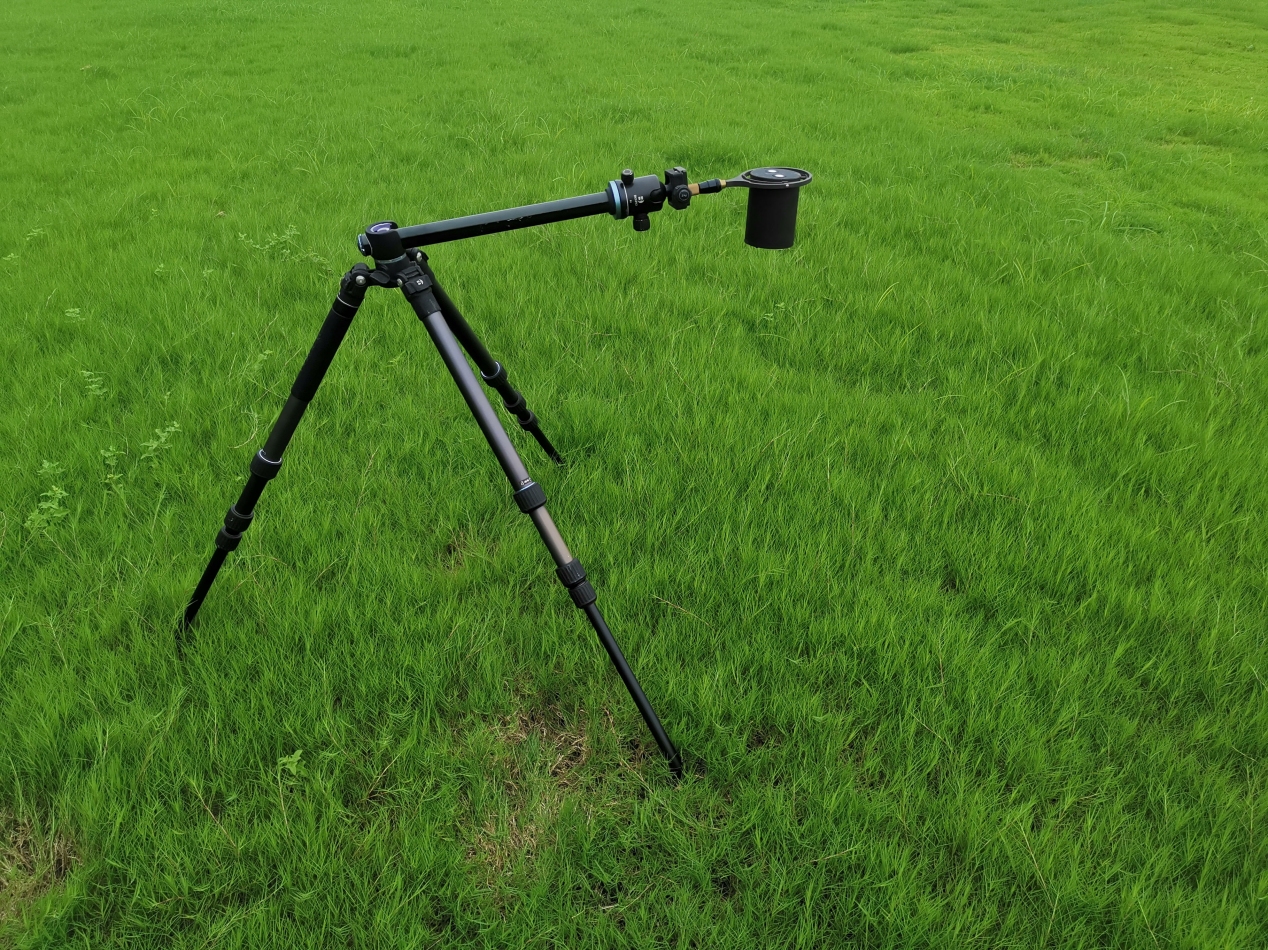
Photograph of the reflectance measurements conducted using the VCR sensor.

To verify the accuracy of the sensor for full-day measurements, the spectral reflectance of Bermuda grass was measured using the VCR sensor from 8:00 to 17:00 under clear sky conditions (with occasional clouds after 15:00) (**Figure 5**). The solar altitude ranged from 23.0° to 74.5°. The VCR sensor was positioned 50 cm above the vegetation, and measurements were taken once per minute while recording the solar altitude.

## 3 Results and discussion

### 3.1 Analysis of calibration results for the VCR sensor

#### 3.1.1 Calibration analysis of TPL0102 resistance values

**Figure 6:**
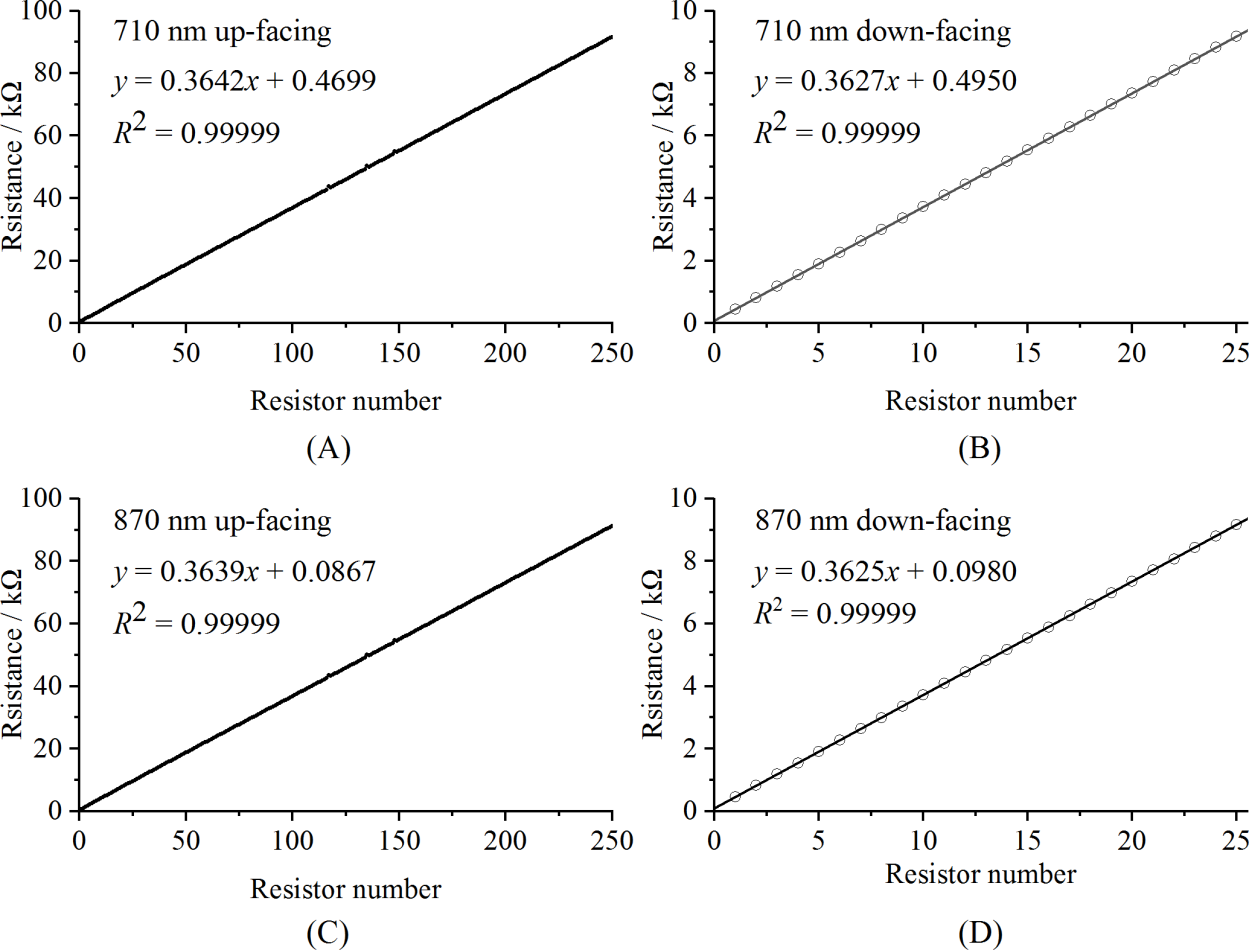
Calibration curves of the TPL0102 for the different optical units: **(A)** 710 nm up-facing optical unit, **(B)** 710 nm down-facing optical unit, **(C)** 870 nm up-facing optical unit, and **(D)** 870 nm down-facing optical unit.

The calibration results of the resistance values of the TPL0102s are shown in **Figure 6**. As can be seen, the resistance values of the TPL0102 in all of the optical units have a strong linear relationship with the resistor number, with *R* ^2^ values reaching 0.99999.

#### 3.1.2 Calibration analysis of OPT101 dark currents in each optical unit

**Figure 7:**
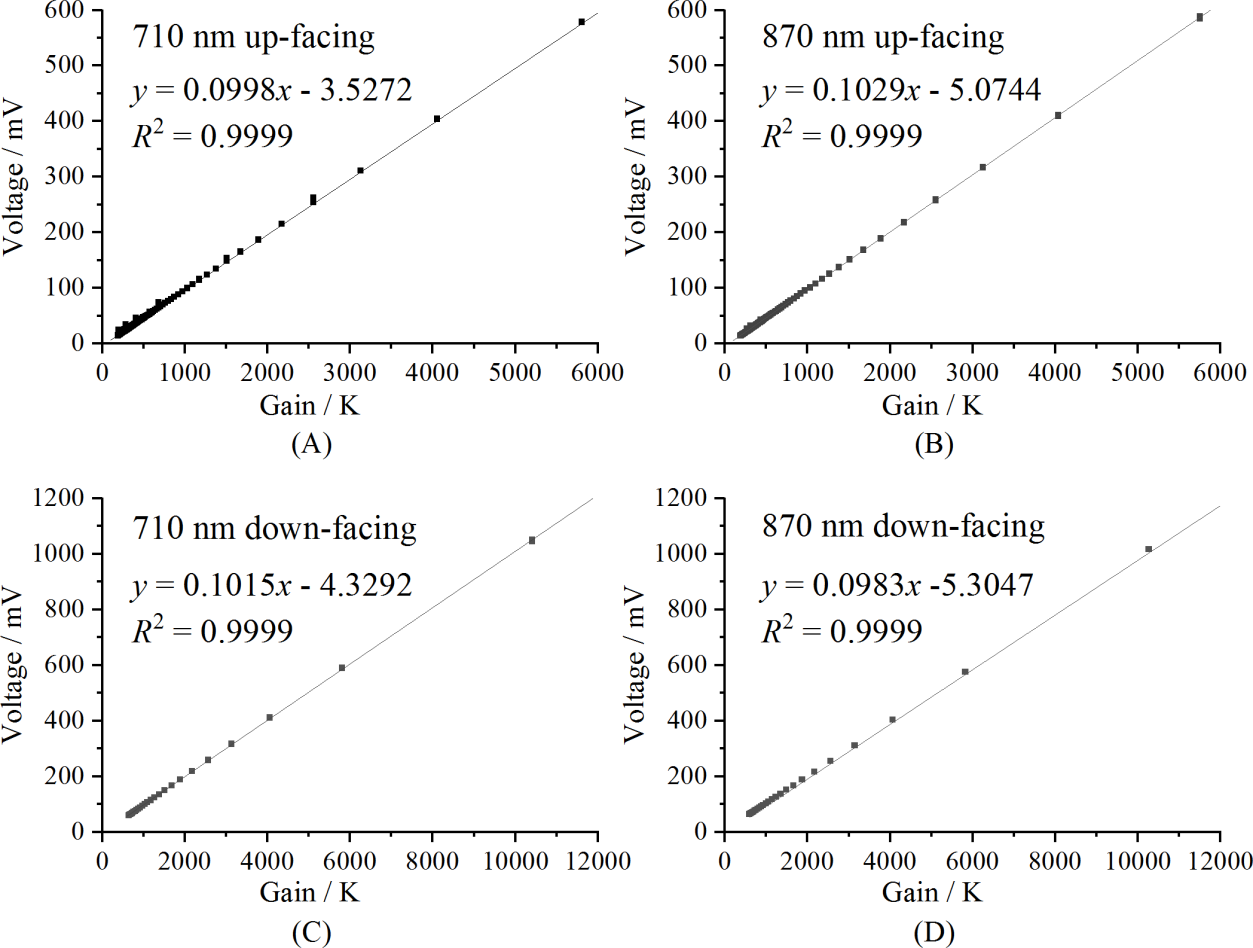
Calibration curves of the voltage generated by the dark current for each optical unit: **(A)** 710 nm up-facing optical unit, **(B)** 870 nm up-facing optical unit, **(C)** 710 nm down-facing optical unit, and **(D)** 870 nm down-facing optical unit.

The calibration results of the dark currents of the OPT101 in each optical unit are shown in **Figure 7**. As illustrated, there is a strong linear relationship between the dark current and the gain of the circuit, with an *R* ^2^ value of 0.9999.

#### 3.1.3 Calibration analysis of the sensor reflectances

**Figure 8:**
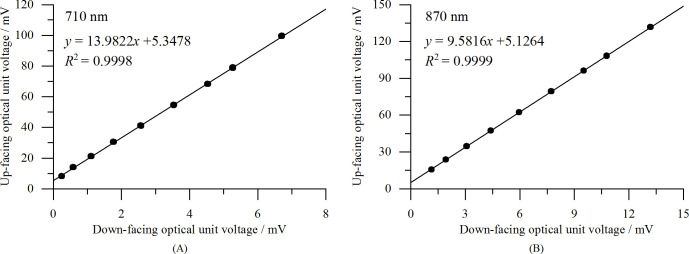
Calibration curves of the output voltages for the down-facing optical units with a gain of 1 K: **(A)** 710 nm, and **(B)** 870 nm.

We used an integrating sphere to calibrate the sensor’s output voltages, thereby achieving reflectance calibration for the VCR sensor. The results of the voltage calibration are shown in **Figure 8**. As can be seen, the down-facing output voltage and the up-facing output voltage at the same wavelength exhibit a strong linear relationship, with *R* ^2^ values of 0.9998 at 710 nm and 0.9999 at 870 nm.

#### 3.1.4 Influence of solar elevation on reflectance measurements by the VCR sensor and the construction of its correction model

**Figure 9:**
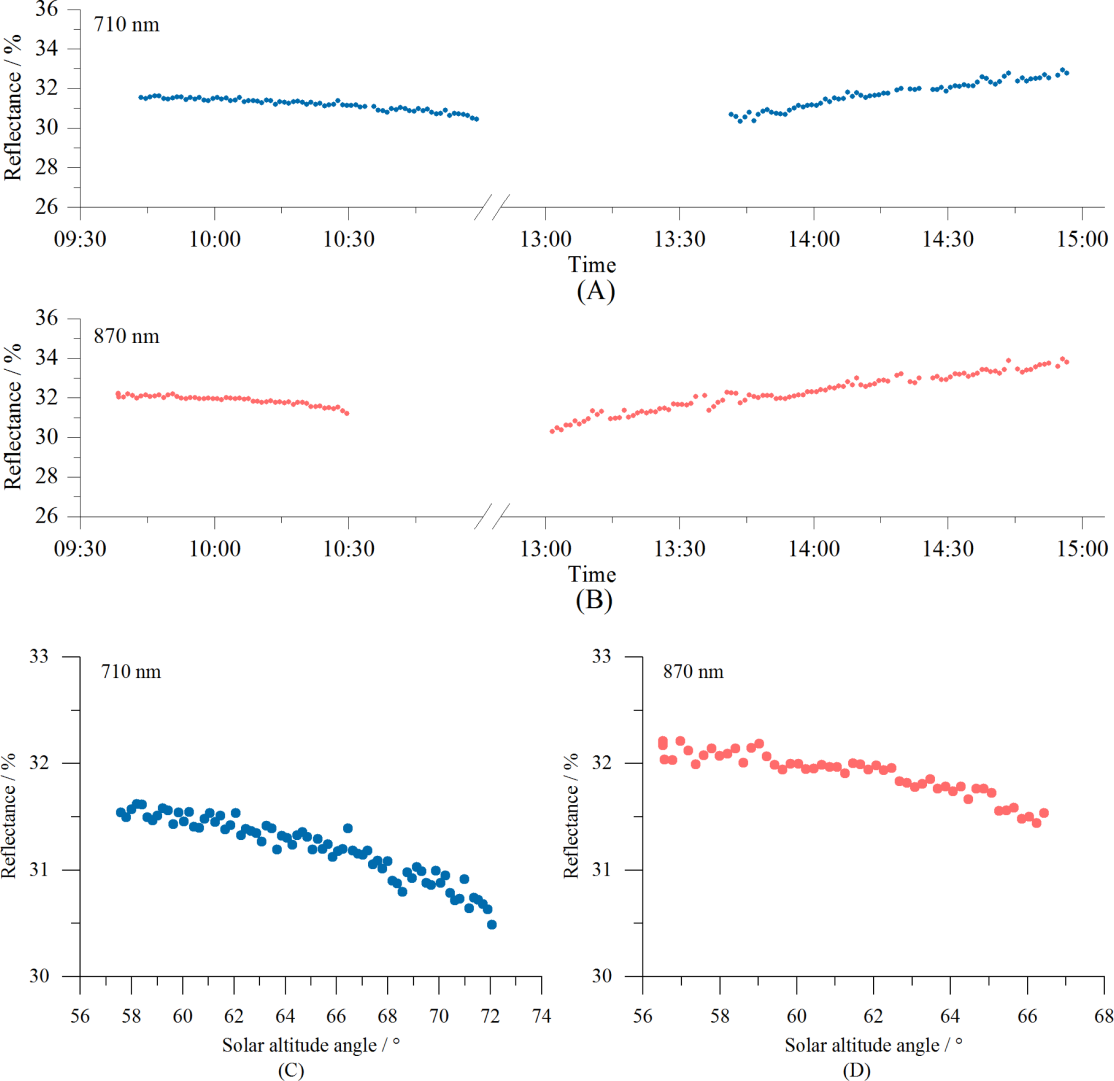
Diurnal variations in the reflectance at 710 nm **(A)** and 870 nm **(B)** measured by the VCR sensor using a 30% reflectance gray scale board. Reflectance changes at 710 nm **(C)** and 870 nm **(D)** as a function of the solar elevation angle.

A 30% reflectance gray panel was used as the monitoring target to examine the variations in the reflectance measured by the calibrated VCR sensor throughout the day. Due to limitations imposed by the distance between the sensor and the gray panel, the sensor’s shadow entered the FOV of the 710 nm optical unit between 11:00 and 13:40, while its shadow entered the FOV of the 870 nm optical unit between 10:30 and 13:00. Consequently, these data points were removed during the preprocessing. The results are shown in **Figure 9**. As shown, the reflectance measurements show a distinct trend throughout the day, with lower values occurring closer to noon (**Figures 9A, B**). Specifically, the reflectance decreases nonlinearly as the solar elevation angle increases (**Figures 9C, D**). The experimental results indicate that the cosine corrector alone cannot fully eliminate the nonlinear influence of the solar elevation on reflectance measurements made by spectral sensors. Therefore, to improve measurement accuracy, it is necessary to develop a solar elevation correction model.

Sky light can be categorized into two main types: sky-scattered light and direct sunlight. Sky-scattered light is diffuse and not affected by the solar elevation angle. As the solar elevation angle increases, the angle of the direct sunlight entering the sensor’s optical window decreases, allowing more light to pass into the sensor. Under clear weather conditions, the proportion of the direct sunlight in the total sky light can reach 80–90%. However, when the solar elevation angle is zero (the Sun at the horizon), all of the light in the sky is diffuse, with no direct sunlight, making the proportion of direct light 0%. To model the variations in the proportions of direct and diffuse light with changes in the solar elevation, a sine function can be used. To ensure that the proportion of direct sunlight is 90% at a solar elevation angle of 90° and 0% at an elevation of 0°, the proportion of direct sunlight (PD) can be expressed as PD=sin(0.7128θ) , where *θ* is the solar elevation angle. The corresponding proportion of diffuse light (PS) is expressed as PS=1−sin(0.7128θ) . This model effectively captures the gradual increase in direct sunlight as the sun rises and the predominance of diffuse light when the sun is low. The voltage value measured by the sensor’s up-facing optical unit (*U_U_*), which represents the light intensity, can be separated into two components: the voltage converted from the diffuse light, expressed as $U_{U}\cdot PS$ and the voltage converted from the direct sunlight, expressed as $U_{U}\cdot PD$ . The direct sunlight can be decomposed into two vectors. One portion follows a vertically downward path, perpendicular to the optical window of the sensor. This portion of the light enters the sensor, and the measured voltage $U_{U}\cdot PD$ is generated from this light. The other part of the sunlight travels horizontally, parallel to the sensor’s optical window, and does not enter the sensor. Based on the vector decomposition, the intensity of the direct sunlight before decomposition can be determined using the formula $\frac{U_{U}\cdot PD}{\sin\theta }$ . Thus, by taking Equation 4 into consideration, the following solar elevation correction model for reflectance can be derived (Equation 5).


(5) $$\rho_{\lambda ,\theta }=\frac{f\left(U_{D}/G_{D}\right)}{\frac{U_{U}\cdot PS}{G_{U}}+\frac{U_{U}\cdot PD}{G_{U}}\cdot \frac{1}{\sin\theta }}=\rho_{\lambda }\cdot \frac{1}{1-\sin\left(0.7128\theta \right)+\frac{\sin\left(0.7128\theta \right)}{\sin\theta }}$$

where $\rho_{\lambda ,\theta }$ is the reflectance in wavelength *λ* when the solar elevation is *θ* (°). The meanings of the other symbols are the same as in Equation 4.

**Figure 10:**
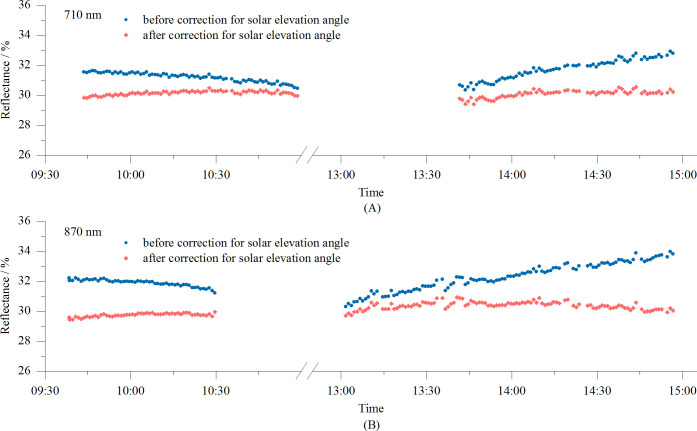
Diurnal variations in reflectance measured by the VCR sensor before and after solar altitude correction: **(A)** 710 nm, and **(B)** 870 nm.

The reflectance measured using the VCR sensor can be corrected using Equation 5 to reduce the impact of the solar altitude on the measurements, enabling all-day operation of the sensor. The corrected results for the data presented in **Figure 9** are shown in **Figure 10**. It is evident that after correction, the diurnal variations in the reflectance at 710 nm and 870 nm are mostly stable, indicating an excellent correction effect.

### 3.2 Analysis of accuracy and stability

#### 3.2.1 Accuracy of standard reflectance gray board measurements

**Figure 11:**
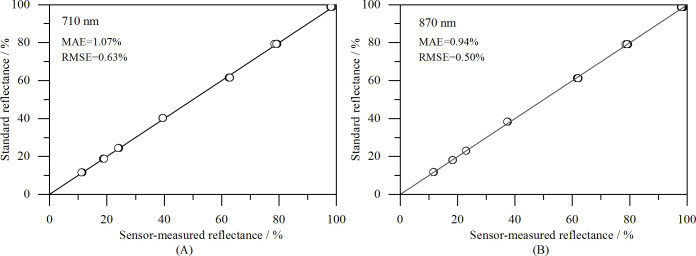
Comparison of measured reflectance and standard reflectance: **(A)** 710 nm, and **(B)** 870 nm.

The accuracy of the VCR sensor measurements was tested using seven gray boards with known reflectance values. The root mean square error (RMSE), mean absolute error (MAE), and 1:1 plot of the measured versus standard reflectance were used to assess the accuracy of the measurements. The results shown in **Figure 11** indicate that the measured reflectance values closely matched those of the gray scale boards. The MAE and RMSE for the 710 nm measurements were 1.07% and 0.63%, respectively, and those for the 870 nm measurements were 0.94% and 0.50%, respectively, demonstrating a high measurement accuracy.

#### 3.2.2 Accuracy of vegetation reflectance measurements

**Figure 12:**
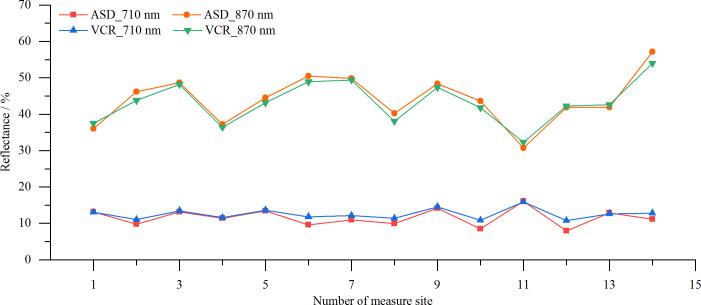
Reflectances of different vegetation canopies measured by the VCR sensor and ASD spectrometer.

The VCR sensor and ASD spectrometer were used to simultaneously measure the spectral reflectance of vegetation canopies at 14 sites. The results are shown in **Figure 12**. The reflectance values at 710 nm and 870 nm measured by the VCR sensor closely matched those measured by the ASD spectroradiometer. The slight deviations observed in some measurements may be due to inconsistency in the vegetation coverage within the fields of view of the VCR sensor and the ASD spectroradiometer.

#### 3.2.3 Accuracy and stability of all-day measurements of vegetation reflectance

**Figure 13:**
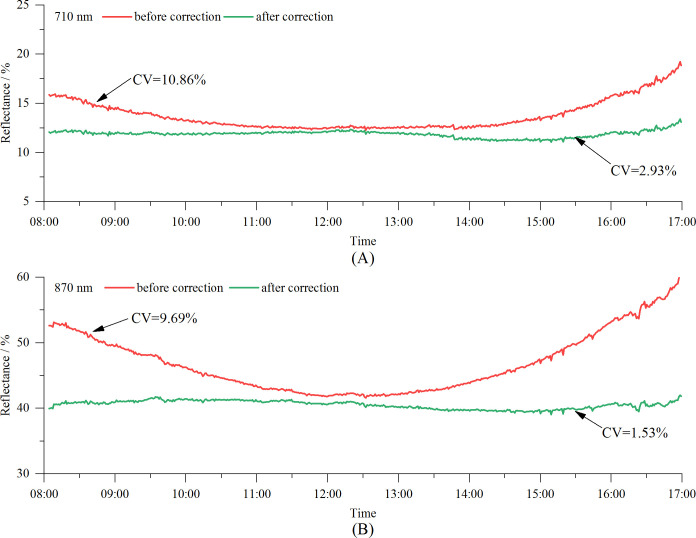
Diurnal variations in spectral reflectance of Bermuda grass measured by the VCR sensor before and after solar altitude correction: **(A)** 710 nm, and **(B)** 870 nm.

The VCR sensor was used to measure the reflectance of Bermuda grass throughout the day. The measured reflectance values were corrected for the solar elevation using Equation 5. The coefficients of variation (CVs) of the reflectances at 710 nm and 870 nm were calculated both before and after the solar altitude correction. The results are shown in **Figure 13**. As can be seen, after correcting for the solar altitude the reflectance measurements remained relatively stable, effectively eliminating the influence of the solar altitude on the results. The all-day reflectance at 710 nm changed from 12.3–19.2% to 11.1–13.4%, and the CV decreased from 10.86% before correction to 2.93% after correction. For the 870 nm all-day reflectance, the values changed from 41.6–60.3% to 39.0–42.0%, and the CV decreased from 9.69% before correction to 1.53% after correction. It can also be seen from the graph that after 16:00, when the solar elevation angle dropped below 23°, the effectiveness of the solar elevation correction model in mitigating the impact of the solar elevation on the measurements began to decline.

### 3.3 Discussion

The spectral reflectance is crucial for monitoring vegetation growth and has been extensively utilized in various fields (Pfitzner et al., 2022; Yu et al., 2022). However, the light intensity in different environments varies significantly. For instance, sun-loving plants thrive in stronger light, while shade-loving plants prefer weaker light. Even for the same plant, the light intensity can vary greatly throughout the year and throughout the day. The variability of the light intensity requires the sensor to have a wide light signal gain range. In this study, precision resistors and TPL0102 potentiometers were used to construct the gain circuit of the photodetector, achieving a gain range of approximately 0.2 M to 10 M. Due to the insufficient accuracy of the potentiometer resistance values, calibration was necessary to enhance the sensor’s accuracy. In this study, a 10 µA constant current source was used to calibrate the TPL0102 for each up-facing optical unit, while a 100 µA constant current source was used for the down-facing optical units. The calibration exhibited excellent linearity, with *R* ^2^ values reaching 0.99999. Based on the gain of each optical unit, the dark current model achieved excellent linearity, with an *R* ^2^ value of 0.9999. During the measurements, for the same wavelength, the up-facing and down-facing optical sensing units had different gain values. After subtracting the dark current, the calculated reflectance was more accurate and reasonable (Ni et al., 2018). Otherwise, nonlinear changes would occur.

Reflectance measurements of vegetation using spectrometers or spectral sensors are mostly conducted around noon (Tian et al., 2005; Huang et al., 2007; Chandel et al., 2021), such as from 11:00 to 13:00 or from 10:00 to 14:00. During this period, due to the small changes in the solar altitude, the measured reflectance is relatively stable. However, this limits the operation hours and reduces the efficiency. Additionally, the high temperatures at noon in summer make fieldwork challenging. Therefore, developing reflectance measurement technology that enables sensors to operate throughout the day is crucial. Like other similar sensors, the VCR sensor is equipped with cosine correctors. However, experimental data reveal that the solar altitude still affects the accuracy of the reflectance measurements (**Figure 9**). Specifically, the sensor’s measured reflectance is a function of both the vegetation canopy and solar altitude, with the lowest reflectance occurring at noon and higher reflectance occurring in the morning and afternoon, resulting in a U-shaped pattern throughout the day. This observation is consistent with previous research findings (Rijks, 1967; Davies and Buttimor, 1969). A plausible explanation for this is that variations in the solar elevation alter the angle of incidence of the direct sunlight on the sensor’s optical window, leading to differences in the cosine corrector’s light transmission efficiency across different solar angles. In this study, we developed a solar altitude correction model and verified the model using Bermuda grass as the test subject. The results demonstrate that after correction, the all-day reflectance measured became much more stable. The CV of the reflectance at 710 nm decreased from 10.86% to 2.93%, while the CV at 870 nm decreased from 9.69% to 1.53%, indicating that the VCR sensor accurately measured the reflectance throughout the day under clear sky conditions. In future research, we will test the sensor on more vegetation to further verify the sensor’s performance.

The characteristics of the VCR sensor developed in this study, along with those of other comparable sensors, are summarized in **Table 1**.

**Table 1:** The characteristics of the selected sensors.

Sensor/Spectrometer	Bands	Size	Portable	Capable of all-day measurements	Capable of batch measurements	Application Field
Sensor developed in this study	710 and 870 nm	71 × 71 × 99 mm	Yes	Yes	Yes	Scientific research/industrial application
CGMD (Ni et al., 2018)	720 and 810 nm	44 × 44 × 52 mm	Yes	Not reported	No	Scientific research/industrial application
Optical sensor for monitoring chlorophyll content (Cui et al., 2009)	610 and 1220 nm	105 × 54 × 124 mm	Yes	No	No	Scientific research/industrial application
FieldSpec FR spectroradiometer	350–2500 nm	127 × 368 × 292 mm	No	Yes Frequent calibration using a white reference panel is required	No	Scientific research

## 4 Conclusions

In this study, we successfully developed a vegetation canopy reflectance sensor that operates at spectral bands of 710 nm and 870 nm, has a 28°FOV and is powered by two 14500 rechargeable batteries, making it highly suitable for field applications. The sensor features Wi-Fi data transmission and a photoelectric conversion circuit with a gain range of 0.2 M to 10 M, ensuring stable and accurate measurements under varying light conditions. A solar altitude correction model was constructed to further enhance the measurement accuracy throughout the day. The sensor’s performance was validated using seven standard reflectance gray scale boards, and MAEs of 1.07% and 0.94%, and RMSEs of 0.63% and 0.50% were achieved for 710 nm and 870 nm, respectively. Field experiments conducted at 14 vegetation sites demonstrated a close agreement between the reflectance values obtained by the VCR sensor and an ASD spectroradiometer in both spectral bands. Additionally, the VCR sensor’s ability to measure the reflectance throughout the day was assessed using Bermuda grass on a clear day (08:00–17:00; solar altitudes of 23.0–74.5°). After applying the solar altitude correction, the reflectance range at 710 nm narrowed from 12.3–19.2% to 11.1–13.4%, and the CV decreased from 10.86% to 2.93%. At 870 nm, the reflectance range narrowed from 41.6–60.3% to 39.0–42.0%, and the CV decreased from 9.69% to 1.53%. In summary, this research provides a foundational tool for obtaining vegetation growth information, with potential applications in improving the efficiency of agricultural and grassland production.

## Data Availability

The raw data supporting the conclusions of this article will be made available by the authors, without undue reservation.
